# Institutional Review Board Assessment—Balancing Efficiency and Quality

**DOI:** 10.31486/toj.19.0075

**Published:** 2020

**Authors:** Stephen J. Rosenfeld

**Affiliations:** Freeport Research Systems, LLC, Freeport, ME

**Keywords:** *Decision making–organizational*, *ethics–research*, *ethics committees–research*, *process assessment–health care*, *quality improvement*

## Abstract

**Background:** Satisfactory measurements of the quality of institutional review board (IRB) reviews and services continue to be elusive. For evaluative purposes, the review process can be separated into two parts: the administrative functions that support the review board and the review board's decisions.

**Methods:** Administrative performance and board decision-making lend themselves to very different measures of quality. In particular, administrative performance is amenable to measures of process efficiency and correctness, while board decisions require a thoughtful consideration of the meaning of quality in the context of the ethical review of research.

**Discussion:** In the evaluation of administrative process, simple numbers such as mean or median time from submission to determination allow for easy comparison between IRBs, but their use means foregoing the opportunity for nuanced assessment and continuous improvement. The full distribution of measured values would indicate whether the IRB takes longer with complex studies or with certain categories of studies. An analysis of outliers would give the IRB an opportunity to assess particularly problematic areas. While such measures and analyses are not easily standardized or shared, they would be useful within the IRB, and one measure of quality would be whether an IRB had procedures in place for routinely conducting such analyses. In the evaluation of decision quality, at a minimum, IRBs should be held to a measure of the consistency of their decisions. Consistency should be used as an internal quality measure. A potential indicator of quality would be the existence of tools and processes for routinely monitoring the consistency of decisions and requiring clear rationale for inconsistencies.

**Conclusion:** Ongoing quality assessment and continuous improvement in decision-making are likely to require committed resources, but such a commitment will be necessary to provide balance to the impetus to improve efficiency and administrative performance.

## INTRODUCTION

Recent changes to federal regulations governing clinical research require most multisite trials to now use a single institutional review board (IRB) for ethical review. Before this requirement, investigators at each site typically relied on the IRB at their institution, but now a single IRB must be explicitly chosen and identified. Because the IRB can have a significant impact on the conduct of the study and on the protections afforded to research participants, the choice of an IRB should be made based on its ability to support the study and the quality of its decisions. However, the IRB community has struggled to develop meaningful measures to assess the quality of IRB review. The reasons for this difficulty include the complexity and variability of the review process, the mission of the IRB, and the subjective nature of IRB decisions.

For evaluative purposes, the review process can be separated into two parts: the administrative functions that support the review board and the review board's decisions. In practice, these two domains do not operate independently; board decisions can be compromised if the information in front of the board is incomplete or not timely, and poorly articulated or poorly justified board decisions can lead to unnecessary communication and delays. Nonetheless, administrative performance and board decision-making lend themselves to very different measures of quality. In particular, administrative performance is amenable to measures of process efficiency and correctness, while board decisions require a thoughtful consideration of the meaning of quality in the context of the ethical review of research (Table).

**Table.** Measures of Institutional Review Board Quality

Domain	Measure	Proposed Process or Metric
Processes	Time to event (eg, review, final determination, approval of final consent version)	Standard definition for all event triggers
		Ongoing review of outliers
		Ongoing review of metric distributions
		Complexity score
	Compliance	With recordkeeping requirements
		With requirements for minutes
		Competencies on roster
		Compliance with the 45 CFR §46.111 criteria for approval
	Consent	Inclusion of required elements of consent
		Review of the circumstances under which consent is sought
	Error rates	Standards for error attribution
Decision-making quality	Consistency	Consistency with past determinations for similar studies or research contexts
		Justification for inconsistencies
	Membership and meetings	Alignment of meeting participants and reviewers with context of specific studies being reviewed
		Appropriate participation of members and consensus seeking
		Member surveys or interviews
	Consent	Understandability, accessibility, and utility of the consent form to actual participants
	Review	Adequacy of minutes to support board decision-making and quality assurance

## ADMINISTRATIVE PERFORMANCE MEASURES

Because they are easily measured and compared, metrics based on performance of the entire IRB apparatus, not just the review board itself, are widely used. Such measures include the time to review and the number of administrative errors (eg, approved informed consent forms with incorrect headers or dates). This focus on measures of process has been reinforced by the independent IRB industry, which, until the last decade, consisted of dozens of companies that reviewed studies that were generally routine or involved interventions for which the risks were well characterized. Because of their routine nature, such studies rarely led to challenging board discussions and decisions and did not require that research risks and potential benefits be assessed de novo in terms of ethical principles. Commercial independent IRBs competed for industry clients on the basis of their process performance, responsiveness, and ease of submission. The commercial nature of these companies also allowed them to devote more resources to administrative process than were typically available to academic institutions. The example set by independent IRBs had a salutary effect on the entire IRB industry because their performance raised investigator and sponsor expectations for IRB performance in general, led to a greater resource commitment from institutions, and generally improved the administrative performance of IRBs across the board.

Administrative performance measures such as review time have been adopted by the Association for Accreditation of Human Research Protection Programs, which requires submission of metrics from accredited organizations and provides benchmarks for such organizations as a benefit of accreditation.^1^

While beneficial for the IRB community as a whole, administrative performance measures can be deceptive if used across individual IRBs or companies. Measures of turnaround time are completely dependent on what events trigger the beginning and end of the period being measured, and no accepted standards exist for such events. In particular, ethical review is typically only one of a series of reviews that an institution must do before committing its people, resources, and reputation to a study, and the ethical review may be contingent on some or all of these other reviews. Even at commercial IRBs, practices can vary as to whether the clock starts at receipt of an application, at staff assessment that the application is complete, or at board member review to confirm completeness. Last, IRBs have no complexity score or other correction for complexity that is analogous to the acuity score corrections used by hospitals when reporting measures of clinical performance. It would be surprising if IRBs at large academic medical institutions did not have longer review times based on the complexity or risk level of their submissions compared to independent IRBs or community hospitals.

The process of IRB review is necessarily complex, and documents and notices of IRB decisions typically pass through many hands. Even if an IRB's decision-making is sound, errors in these handoffs can occur. Measures of error rates are neither straightforward nor comparable. While identifying errors may be easy, attributing responsibility for errors is more difficult. Was there an error in the submission documents? Should that error have been detected by IRB staff and therefore reported in the IRB's error statistics, or should the IRB treat all submissions as complete and consider errors in submissions to be the responsibility of the sponsor or investigator? If the board requires more information before making a determination, was that an error in the submission and therefore the resulting delay should not be reflected in the IRB's timing metrics? The IRB must also decide what level of resources to commit to categorizing errors, given that these resources might be better used to simply fix the errors or support the review process itself.

The seduction of a simple number, such as mean or median time from submission to determination, also undermines real assessment of IRB performance. Simple numbers allow for easy comparison between IRBs, but their use means foregoing the opportunity for more nuanced assessment and continuous improvement. The full distribution of measured values would indicate whether the IRB takes longer with more complex studies or with certain categories of studies, such as those involving a medical device. Similarly, an analysis of outliers would give the IRB an opportunity to assess particularly problematic areas. While such measures and analyses are not easily standardized or shared, they would be particularly useful within the IRB, and one measure of quality would be whether IRBs had procedures in place for routinely conducting such analyses.

## MEASURES OF BOARD DECISION QUALITY

Determinations about the ethical acceptability of research involving human participants is the mission-driven product of the IRB. Despite this centrality, the quality of these decisions is difficult to assess. The challenge of ethical decision-making was described in the Belmont Report,^2^ which reflected on the challenge of applying existing codes of research conduct, starting with the Nuremberg Code^3^:

The codes consist of rules, some general, others specific, that guide the investigators or the reviewers of research in their work. Such rules often are inadequate to cover complex situations; at times they come into conflict, and they are frequently difficult to interpret or apply. Broader ethical principles will provide a basis on which specific rules may be formulated, criticized and interpreted.^2^

Three principles, or general prescriptive judgments, that are relevant to research involving human subjects are identified in this statement. Other principles may also be relevant. These three are comprehensive, however, and are stated at a level of generalization that should assist scientists, subjects, reviewers and interested citizens to understand the ethical issues inherent in research involving human subjects. These principles cannot always be applied so as to resolve beyond dispute particular ethical problems. The objective is to provide an analytical framework that will guide the resolution of ethical problems arising from research involving human subjects.^2^

Thus, the role of the IRB is to apply Belmont's “analytical framework,” and the report explicitly acknowledges that such application “cannot always be applied so as to resolve beyond dispute particular ethical problems.” If either codes of research conduct or Belmont's three principles—respect for persons, beneficence, and justice—were able to definitively answer complex ethical questions, there would be no need to impanel a committee to do so; such a determination could presumably be made by an individual well trained in the principles. While some ethical questions are straightforward, many of the questions that arise in the research setting involve balancing the rights and welfare of individuals with the societal benefit of the scientific agenda, and this balancing is necessarily not a matter of fact alone but also of societal context and individual judgment. In fact, the first role of the IRB is not to decide if a particular research project satisfies this balance but rather to determine if the balance is *sufficiently satisfied* that it is appropriate to offer potential participants (individuals who typically do not have the training or dispassion to objectively judge the potential harms and benefits of the research) the opportunity to make their own decisions to participate.

Further, there is a misunderstanding that the IRB is the primary mechanism for the protection of human subjects involved in research. 45 CFR §46,^4^ known as the Common Rule, is titled “Protection of Human Subjects,” as is 21 CFR §50,^5^ the US Food and Drug Administration regulations governing IRBs. As noted above, the actual charge to the IRB is to assess the *ethical acceptability* of research. Such an assessment is not a one-time event but needs to be ongoing, as accumulating knowledge allows continued refinement of the estimated risk of harms, possible benefits, and scientific value of the study. This role was made explicit in a 2003 report from the National Academies.^6^ That report described human research participant protection programs as “a system of interdependent elements that involve the research organization, the IRB, the investigators, the sponsors, and most importantly, the volunteer participants,” and described four essential functions of such programs:

1. Comprehensive review of protocols (including scientific, financial conflict of interest, and ethical reviews)
2. Ethically sound participant-investigator interactions
3. Ongoing (and risk-appropriate) safety monitoring throughout the conduct of the study
4. Quality improvement and compliance activities

Note that the IRB is explicitly charged with only one of these activities: comprehensive ethical review. The report noted that IRBs have increasingly been tasked with duties not directly related to ethical assessment and recommended that the role and mission be clarified and refocused by renaming them research ethics review boards.

IRB determinations are only one step in a very complex system to protect human subjects and the step possibly furthest removed from the actual, day-to-day conduct of research where harms are realized. The mistaken view that IRBs are the primary mechanism to protect research participants against harms has consequences for the debate about IRB quality.^7^ In particular, such a view suggests that IRBs should be assessed against the impact of their review on reducing actual harms to research participants rather than against the completeness and integrity of the review itself. In fact, IRBs are charged with determining that “Risks to subjects are minimized” and that “Risks to subjects are reasonable in relation to anticipated benefits.”^8^ This language explicitly recognizes that participants are expected to face risks of harm and that the role of the IRB is not to eliminate such risks but to assess whether they are ethically justified. In practice, risks are experienced at the level of individual protocol activities and participant-investigator interactions and are mitigated through ongoing safety monitoring, although the IRB may have a role in reacting to both of these in its role of ensuring ongoing balance of benefits and risks of harm. In other words, reduction in harms is not the same as ethical acceptability, which requires that risks of harms be minimized *and* that risks be appropriately balanced by the possibility of broader benefits.

In addition, assessing individual harms consequent to individual IRB review has practical limitations, in that it would be impossible or very difficult to create a comparator in which participants were exposed to research harms without review. Last, any such assessment would take place within the context of a research enterprise broadly subject to ethical review; eliminating or controlling for the decisions of a single IRB is likely to greatly underestimate the persistent impact of the IRB system as a whole.^9^

Some traditional research studies are obviously unethical in concept, but in today's mature research environment, these are few and far between, and most IRBs would be expected to be able to detect such studies as a minimal level of competence not as a measure of decision-making quality. In contrast, the charge to minimize risks and ensure reasonable balance means that IRBs have a role in the refinement and improvement of studies so that potential participants are not faced with accepting unnecessary risks as a cost of participation. In this context—the refinement of studies—variability in IRB decisions has been reported.^10^ Such variability can be a cause of concern and wasted resources as sponsors and investigators scramble to meet sometimes conflicting requirements from different IRBs. As noted above, some measure of variability is expected, given both the nature of the ethical problems an IRB must resolve and the reality that the IRB is composed of a small number of individuals with different personal experiences^11^; it is when this variability is arbitrary that it is unjustified. At a minimum, IRBs should be held to a measure of the consistency of their decisions, with the caveat that variability is actually a sign of good decision-making if it is based on difference of fact and if it is well explained. Consistency should be used as an internal quality measure, but there is no benchmark, and inappropriately consistent decisions are as troubling as arbitrarily inconsistent decisions. A potential indicator of quality would be the existence of tools and processes for routinely monitoring the consistency of decisions and requiring clear rationale for inconsistencies.

Consistency can only be assessed against similar research. Increasingly, IRBs are faced with novel research driven by evolving technology (eg*,* full genome sequencing, pervasive data, CRISPR) or new ethical situations driven by evolving social norms (eg*,* increasing awareness of structural injustice). In such circumstances, IRBs have a role in establishing precedent and informing the broader societal debate. In these situations, there is little justification for allowing the rationale behind IRB decisions to be left unstated or unshared, and another measure of a high-quality IRB should be a willingness to transparently explain and argue the correctness of its decisions.

Decisional quality has aspects beyond consistency and transparent reasoning. The regulations have specific requirements for what, at a minimum, the IRB should consider in making the decision to approve a research proposal or not. Compliance with these requirements has been proposed as a measure of quality, specifically whether the IRB has explicitly discussed the individual elements of 45 CFR §46.111.^12^ Such a proposal highlights the dangers of process measures, including those described above; they can become checklists that do not reflect actual substantive review. Nonetheless, periodic retrospective review to confirm that the IRB has considered and discussed specific elements, such as selection of subjects, in studies where these elements are not straightforward, would be a valuable measure of an internal quality assurance program.

Another measure of the ability of the IRB to make appropriate decisions is the qualifications of its members. According to the regulations,

The IRB shall be sufficiently qualified through the experience and expertise of its members (professional competence), and the diversity of its members, including race, gender, and cultural backgrounds and sensitivity to such issues as community attitudes, to promote respect for its advice and counsel in safeguarding the rights and welfare of human subjects. The IRB shall be able to ascertain the acceptability of proposed research in terms of institutional commitments (including policies and resources) and regulations, applicable law, and standards of professional conduct and practice. The IRB shall therefore include persons knowledgeable in these areas.^13^

An ongoing debate in the IRB community is whether the IRB should include individuals sufficiently knowledgeable about the science involved in every research project to definitively address the project's potential value without a separate scientific assessment, but the members of the IRB (or those members augmented by consultants) must, at a minimum, be able to address the research context and the scientific value sufficiently to address the first two criteria for approval: (1) “Risks to subjects are minimized” and (2) “Risks to subjects are reasonable in relation to anticipated benefits, if any, to subjects, and the importance of the knowledge that may reasonably be expected to result.”^8^

In addition, the IRB must have members who can address the third criterion for approval:

Selection of subjects is equitable. In making this assessment the IRB should take into account the purposes of the research and the setting in which the research will be conducted. The IRB should be particularly cognizant of the special problems of research that involves a category of subjects who are vulnerable to coercion or undue influence, such as children, prisoners, individuals with impaired decision-making capacity, or economically or educationally disadvantaged persons.^8^

These concerns suggest that the board roster and the match between individuals participating in a meeting and the scope of the studies reviewed at that meeting should also be considered a quality measure. Further, the roster or record of attendance is not sufficient to demonstrate that the different perspectives required by regulation actually have a voice. Board discussions are frequently dominated by a few voices because of authority, expertise, or personality.^14^ Ideally, decisions should be made after consensus is sought, even if determinations are not unanimous. Such decision-making processes are difficult to measure; in many cases, lack of discussion may simply reflect the straightforward or conventional nature of the research proposal. Reducing appropriate participation to a single number is difficult; rather, the IRB should have processes that monitor participation, discussion, and whether certain voices are systematically absent from the debate. One way to collect this information is to review meeting minutes or recordings; another way is to regularly survey board members to ask if they feel their perspectives are heard.

As noted above, any assessments of IRB decision-making quality require a comprehensive record of IRB activities. IRBs are required to maintain

Minutes of IRB meetings, which shall be in sufficient detail to show attendance at the meetings; actions taken by the IRB; the vote on these actions including the number of members voting for, against, and abstaining; the basis for requiring changes in or disapproving research; and a written summary of the discussion of controverted issues and their resolution.^15^

Official minutes or other records must be maintained not only for regulatory compliance but also because records of past determinations and their rationales create a growing resource for IRBs to learn from and for their decisions to evolve with changing societal mores. However, concerns about privacy and liability have led to a minimalist approach to minutes. In particular, limiting minutes to recording discussion of controverted issues and “the basis for requiring changes in or disapproving research” robs the IRB of the ability to retrospectively assess the adequacy of decisions that were *not* controverted or disapproving and severely limits the opportunities for quality assurance and continuous improvement.

Last, there is another aspect to the quality of review, even after decisional quality is assured. Once an IRB has determined that the balance of risks of harm and potential benefits is sufficient to allow individuals to make the decision whether or not to participate, their next task is to ensure that such a decision is fully informed and freely made.^16^

The pre-2018 Common Rule required the following:

- Except as provided elsewhere in this policy, no investigator may involve a human being as a subject in research covered by this policy unless the investigator has obtained the legally effective informed consent of the subject or the subject's legally authorized representative.
- An investigator shall seek such consent only under circumstances that provide the prospective subject or the representative sufficient opportunity to consider whether or not to participate and that minimize the possibility of coercion or undue influence.
- The information that is given to the subject or the representative shall be in language understandable to the subject or the representative.
- No informed consent, whether oral or written, may include any exculpatory language through which the subject or the representative is made to waive or appear to waive any of the subject's legal rights, or releases or appears to release the investigator, the sponsor, the institution or its agents from liability for negligence.

The 2018 Common Rule adds additional requirements:

- The prospective subject or the legally authorized representative must be provided with the information that a reasonable person would want to have in order to make an informed decision about whether to participate, and an opportunity to discuss that information.
- Informed consent must begin with a concise and focused presentation of the key information that is most likely to assist a prospective subject or legally authorized representative in understanding the reasons why one might or might not want to participate in the research. This part of the informed consent must be organized and presented in a way that facilitates comprehension.
- Informed consent as a whole must present information in sufficient detail relating to the research, and must be organized and presented in a way that does not merely provide lists of isolated facts, but rather facilitates the prospective subject's or legally authorized representative's understanding of the reasons why one might or might not want to participate.

Assessment of how well IRBs manage informed consent forms has focused largely on the explicit elements of consent and whether or not they are included in the approved documents, but the general requirements for consent offer several other potential quality measures that could be collected, tested, and assessed. The simple requirement that no subject be enrolled absent legally effective consent can be delegated to the site or institution through that entity's assurance or other mechanism—legally effective consent can be assessed through the presence or absence of a signed form—and the site would be accountable if consent were not obtained. On the other hand, delegating compliance with the circumstances of consent is more problematic, in that such compliance is not documented by a signature that can be assessed retroactively but can only be assessed by observing the actual context of consent. Further, investigators and study teams operate under a number of administrative and time pressures that are in tension with the regulatory requirements. IRBs have the authority to observe the consent process, and the extent to which they periodically exercise this authority is another potential quality measure.

How the consent form is written, ie, the understandability, accessibility, and utility of the form to actual participants when they make a decision whether or not to enter a study, is rarely formally assessed. Concerns about whether consent forms are too long and serve too many other purposes are longstanding,^6^ and another potential measure of IRB quality is whether the forms approved by the IRB are readable and useful to research participants. Given the study-specific nature of consent forms, such a metric would best be collected in the context of individual studies, perhaps by interviews with or surveys of appropriately sampled research participants.

## CONCLUSION

The current practice of assessing IRBs only on measures of administrative performance is understandable, in that such measures are relatively easy to collect and can be reduced to simple numbers. In contrast, implementing the proposed measures of decisional quality would require an internal quality assurance program to routinely review board decisions and examine outliers, and would require additional staff or take existing staff away from direct review activities. Administrative performance and decisional quality are in tension—the simplest way to streamline the former is to reduce the latter to minimal compliance—and this tension highlights the need for assessment of both so that they remain appropriately balanced. Absent accepted measures of decisional quality, external pressures on IRBs will continue to focus on reducing costs and review time, only offset by the need to maintain minimal compliance. The necessarily subjective nature of ethical review increases the danger of this approach, in that compliance to the letter of the regulations is relatively easy to demonstrate even without meaningful discussion, and attention to compliance without quality undermines the role of the IRB and the purpose of the ethical review system as a whole.

## Notes

This article meets the Accreditation Council for Graduate Medical Education and the American Board of Medical Specialties Maintenance of Certification competencies for Patient Care, Medical Knowledge, and Systems-Based Practice.
